# The Aptamer bi-(AID-1-T) Synergizes with Radiation to Inhibit Proliferation of Human Glioma Cells

**DOI:** 10.3390/pharmaceutics17111442

**Published:** 2025-11-08

**Authors:** Svetlana Pavlova, Ksenia Rubetskaya, Lika Fab, Ekaterina Savchenko, Nadezhda Samoylenkova, Alexander Revishchin, Anastasia Ryabova, Natalia Antipina, Mikhail Galkin, Andrey Golanov, Dmitry Usachev, Alexey Kopylov, Galina Pavlova

**Affiliations:** 1 Institute of Higher Nervous Activity and Neurophysiology, Russian Academy of Sciences, 117485 Moscow, Russia; 2 Federal State Autonomous Institution N. N. Burdenko National Medical Research Center of Neurosurgery of the Ministry of Health of the Russian Federation, 125047 Moscow, Russia; 3 Prokhorov General Physics Institute, Russian Academy of Sciences, 119991 Moscow, Russia; 4 Belozersky Research Institute of Physical Chemical Biology, Lomonosov Moscow State University, 119991 Moscow, Russia; kopylov.alex@gmail.com (A.K.); 5 Department of Medical Genetics, Sechenov First Moscow State Medical University, 119991 Moscow, Russia

**Keywords:** high-grade glioma, cell migration, aptamers, G-quadruplexes, nucleic acids, radiation, anti-proliferative activity, anti-migration activity

## Abstract

**Background:** High-grade gliomas are treatment-resistant and prone to aggressive recurrence. Although radiation therapy is a fundamental treatment, it often fails to eradicate tumors and can enhance the migratory potential of surviving cells, promoting relapse. Anti-proliferative aptamers are novel agents that show promise, but their combination with radiation therapy and their effects on invasive phenotypes require further investigation. **Objectives:** This study evaluated the effects of ionizing radiation on the viability and migration of human glioma cells, both alone and in combination with the anti-proliferative aptamer bi-(AID-1-T). The study aimed to determine whether the aptamer could enhance the efficacy of radiotherapy and counteract ionizing radiation-induced pro-migratory effects. **Methods:** The study was conducted on cell cultures of primary and relapsed human glioma. The effects of combined radiation (single dose of 20 Gy) and the bi-(AID-1-T) aptamer (10 μM) were assessed using the MTS assay, Transwell analysis, immunocytochemistry and transcriptome analysis. **Results:** Ionizing radiation alone reduced proliferation in primary gliomas, but increased proliferation in recurrent cultures. Ionizing radiation also increased migration in both types of gliomas. Combining ionizing radiation with the bi-(AID-1-T) aptamer produced a synergistic effect: it significantly reduced cell proliferation and migration, and suppressed the ionizing radiation-induced migratory enhancement, more effectively than either treatment alone. Transcriptome analysis revealed that combination treatment decreased the expression of pro-proliferative and migratory genes (e.g., *PDPN*, *CDH3*), while increasing the expression of anti-migratory (*RND3*) and pro-apoptotic genes (e.g., *XAF1*, *SEMA3A*). Thus, combination treatment significantly reduces tumor cell proliferation and migration; however, further studies on surviving cells are needed.

## 1. Introduction

High-grade gliomas (HGGs), particularly glioblastoma (World Health Organization (WHO) grade 4), remain one of the most malignant oncological diseases of the central nervous system. Despite advanced treatments, including surgical resection followed by adjuvant radiochemotherapy (most often radiation therapy combined with temozolomide), the median overall survival rarely exceeds 15–20 months, and the 5-year survival remains catastrophically low—less than 7% [1,2,3].

Unfortunately, the extraordinary infiltrative capacity of glioblastoma cells is one of the main factors determining the frequency and inevitability of tumor relapse. Glioblastoma cells are able to migrate along white matter tracts, blood vessels and subarachnoid spaces, penetrating functionally important areas of the brain. This infiltration makes it impossible to completely remove malignant cells surgically without damaging vital brain structures. Regrettably, the remaining cancer cells are resistant to adjuvant therapy [4,5,6,7].

In addition, an alarming phenomenon has been recently noticed in the treatment of glioblastomas. Therapeutic interventions themselves have been found to stimulate migration and invasion of the surviving tumor cells. Despite the extensive research and the development of new therapeutic approaches in recent decades, radiation therapy (RT) remains one of the key treatments of malignant gliomas. Its effectiveness is significantly limited by several biological features of the tumor and technical challenges, which leads to relapse and progression of the disease in most patients [8,9].

Hyperactivation of the epidermal growth factor receptor (*EGFR*) signaling pathway, often due to gene amplification, and inactivation of the *PTEN* tumor suppressor gene are the main drivers of the aggressive phenotype of high-grade gliomas [10,11,12].

Transmembrane proteins and non-coding RNAs may also play an important role in the pathogenesis of glioma. In particular, the transmembrane protein TMEM64 promotes tumor progression through activation of the Wnt/β-catenin pathway, while the long non-coding RNAs LINC00346 and CHASERR are involved in glioma proliferation and migration [13,14,15].

The main factor of tumor resistance to radiotherapy is pronounced intratumoral heterogeneity and the presence of radioresistant cell subpopulations, including glioma stem cells (GSCs). These cells have a pulsed infrequent proliferation, allowing them to escape radiation exposure, which primarily targets only actively proliferating tumor cells. In addition, GSCs have an enhanced capacity for DNA repair and are often found in hypoxic niches. Hypoxia directly reduces radiosensitivity since the formation of lethal DNA damage as a result of radiation depends on the presence of oxygen [16,17,18,19]. In addition, hypoxia induces transcription factors such as *HIF-1α*, Hypoxia Inducible Factor 1, stimulating tumor angiogenesis, invasion, and metabolic adaptation and further exacerbating the problem [20,21,22,23,24].

Standard therapeutic approaches, including radiation therapy, demonstrate limited efficacy. Furthermore, sublethal doses of ionizing radiation can not only select for resistant clones but also directly stimulate the invasive potential of surviving tumor cells, a phenomenon known as “radiation-induced migration and invasion.” Radiation has been shown to activate the proinflammatory transcription factor NF-κB and increase the expression of matrix metalloproteinases (*MMP-2/9*), which degrade the extracellular matrix. Stabilization of HIF-1α in the hypoxic niche, which worsens after irradiation, further enhances the expression of genes associated with invasion [9,25,26].

Besides, invasive glioma growth remains a significant challenge. Tumor cells migrate far beyond the macroscopically visible tumor and the contrast-enhanced zone on magnetic resonance imaging (MRI). It is practically impossible to visualize single migrated cells and target them with a therapeutic dose of radiation without damaging the surrounding healthy brain tissue [9,27,28].

Considering these limitations, strategies for combining RT with targeted agents that might overcome tumor cell resistance while minimizing damage to the surrounding healthy tissues have been actively explored. Of particular interest are aptamers that target specific tumor molecular markers. Aptamers are short, single-stranded oligonucleotides that specifically bind targets with high affinity and selectivity. Their unique properties (small size, relative ease of synthesis and modification, low immunogenicity, and potential ability to penetrate tissues) make them promising candidates for combined therapies with RT [29,30].

Currently, aptamers are most often used in glioma therapy as various conjugates, serving as highly specific delivery systems that transport therapeutic agents across the blood–brain barrier to target cells, thereby minimizing systemic toxicity and enhancing treatment efficacy [31,32,33]. However, a number of aptamers are capable of independently inhibiting tumor cell proliferation and survival by sterically blocking key receptors and signaling pathways, thereby presenting opportunities for developing monotherapeutic drugs based on these molecules [33,34,35,36].

G-quadruplexes (GQs) are a special class of oligonucleotides currently being explored as potential anticancer agents. These guanine-rich oligonucleotides are characterized by enhanced thermodynamic stability and reduced immunogenicity, which results in their highly specific interactions with target proteins [37,38]. One of the promising antiproliferative G-quadruplexes is bi-(AID-1-T), whose target protein in tumor cells is the cytoplasmic form of PARP1 [34]. In a number of cases, failures in glioma therapy are closely associated with the DNA repair system, particularly PARP1, making it a high-priority therapeutic target. A proven strategy to disrupt DNA repair and sensitize tumor cells to radiation and chemotherapy is PARP1 inhibition. Additionally, PARP1 is also involved in the regulation of transcription and activation of several oncogenic signaling pathways, including NF-κB. Therefore, it is hypothesized that a PARP1-specific aptamer would exhibit dual action, enhancing the DNA-damaging effects of radiation and modulating signaling cascades that underlie glioma aggressiveness and radiation-induced invasion [39,40]. This aptamer has shown promising results in the treatment of primary gliomas; however, no clear conclusion could have been done after the treatment of the cell cultures obtained from glioma relapses. In a few cell cultures, activation of migration processes was observed, whereas in some other cells cultured from relapse tumors, an increase in the proliferative activity was detected [35,41]. At the same time, bi-(AID-1-T) did not demonstrate cytotoxicity against normal cells. This selectivity indicates its advantages as an anti-glioma agent compared to other GQ oligonucleotides [41,42,43].

However, similar results can be observed following exposure to many antitumor drugs. Therefore, the combination of drug therapy with radiation may eliminate the shortcomings of drug therapy alone and remains a preferable strategy for glioma treatment. In this paper we investigated the effect of the bi-(AID-1-T) aptamer on the proliferative and migratory activity of human glioma cell cultures obtained from postoperative patient tumors after exposure of these cells to radiation. In addition, we conducted a transcriptome analysis of one of the cultures after the combined cell treatment.

## 2. Materials and Methods

### 2.1. Cell Cultures

All cell cultures were obtained from postoperative material of patients within the biobank of the Federal State Autonomous Institution N. N. Burdenko National Medical Research Center of Neurosurgery of the Ministry of Health of the Russian Federation (Center for Collective Use “Bioresource Collection of Tissue and Cell Cultures of Tumors of the Human Nervous System for Fundamental and Applied Research”), complying with all formal requirements of the Russian Federation. The Ethics Committee of the Burdenko Neurosurgical Institute of the Russian Academy of Medical Sciences approved this study (protocol number 12/2020). All patients provided written informed consent in accordance with the Declaration of Helsinki. The cells were cultured in DMEM/F12 medium (Servicebio, Wuhan, China), supplemented with 10% FBS (Biowest, Nuaille, France), 2 mM L-glutamine (Paneco, Moscow, Russia), and a 1% antibiotic solution (penicillin/streptomycin) (Corning, Corning, NY, USA), at 37 °C and 5% CO_2_. The cells were removed from the culture vessels using Versene (Paneco, Russia) and 0.25% trypsin (Paneco, Russia) solutions. All cell cultures were used within 10 passages.

### 2.2. Treatment with Aptamers

Before use, the aptamers were prepared by incubating a 100 µM solution of the aptamer at 95 °C for five minutes in a buffer solution containing 10 mM KCl, 140 mM NaCl, and 20 mM Tris-HCl, followed by cooling to room temperature. Then, the solution was diluted with culture medium to a concentration of 10 µM and added to cell cultures. Concentration was selected in previous studies [34,35]

### 2.3. Cell Proliferation Assessment by MTS Assay

Human glioblastoma cell cultures were seeded in 96-well plates (Nest, Wuxi, China) at a density of 7000 cells per well. After the cells attached, combinations of aptamers that appeared to be the most effective for assessing cell viability using xCELLigence RTCA were added. Seventy-two hours after the first aptamer was added, one-tenth of the volume of the medium was replaced with MTS reagent (Promega, Madison, WI, USA). After incubating for 90 min at 37 °C and 5% CO_2_, the absorbance was measured at 495 nm using a SPECTROstar Nano (BMG LABTECH, Ortenberg, Germany).

### 2.4. Cell Migration Assessment by Transwell Inserts

Cell migration activity after aptamer exposure was assessed using Transwell inserts with 8 μm pores (Corning Falcon, Corning, NY, USA, 353097). The cells were incubated in a serum-free medium for one hour. Then, 20,000 cells in 350 µL of serum-free medium were transferred into each insert. The corresponding wells contained 700 µL of culture medium with 10% FBS. Twenty hours later, the non-migrated cells on the inner side of the insert were removed, and the migrated cells on the outer side were fixed with 4% paraformaldehyde and stained with Hoechst 33342 (Sigma, Burlington, MA, USA). The number of migrated cells was assessed in 6 non-overlapping fields of view. The ratio of migrated cells to the initial number of cells in the chamber was then determined. Wells without aptamer addition were used as controls.

### 2.5. Immunocytochemistry (ICC)

The cells were planted into culture plates with pre-positioned coverslips (SPL Life Sciences, Pocheon-si, Gyeonggi-do, Republic of Korea) at a density of 3 × 10^4^ cells per coverslip and incubated for 24 h at 37 °C and 5% CO_2_. Then, the cells were fixed with 4% paraformaldehyde and stained with primary antibodies for L1CAM (ThermoFisher, Waltham, MA, USA), Sox2 (Santa Cruz Biotechnology, Dallas, TX, USA), p53 (Abcam, Waltham, MA, USA), CD44 (Invitrogen, Carlsbad, CA, USA), CD133 (Miltenyl Biotec, Bergisch Gladbach, Germany), EGFR (Invitrogen, USA), Nestin (Invitrogen, USA), β-III-tubulin (Abcam, USA) and Caspase 3 (Abcam, USA). Secondary antibodies were labeled with Cy2 (Jackson ImmunoResearch, West Grove, PA, USA) or Cy5 (Jackson ImmunoResearch, USA). The cell nuclei were stained with bisbenzimide Hoechst 33342 (Sigma, USA), dissolved in PBS. The cells were fixed in Mowiol 4–88 (Sigma-Aldrich, Burlington, MA, USA) with 1% DABCO for eight hours at 4 °C, followed by 24 h at room temperature. The samples were analyzed using a Carl Zeiss LSM-710 (Carl Zeiss Microscopy GmbH, Jena, Germany) series confocal microscope. Cell cultures stained with secondary antibodies, not primary antibodies, were used as controls. The average fluorescence intensity of the cells was determined using ImageJ v1.53n software.

### 2.6. Radiation

For each analysis, a culture flask or a 96-well plate was irradiated using a TrueBeam STx linear accelerator (Varian, Palo Alto, CA, USA), which is commonly used in medical practice and receives regular technical maintenance. The phantoms were exposed to a single vertical bremsstrahlung radiation beam with a rated energy of 6 MeV and a dose rate of 600 cGy/min. A field size of 32 × 32 cm ensured uniform radiation of the entire culture. The SSD was 98 cm. The radiation dose was 20 Gy.

To evaluate the combined effects of irradiation and the bi-(AID-1-T) aptamer, a single 20 Gy dose of irradiation was administered. Then, the aptamer was added 72 h later, followed by analysis 72 h after that.

The control cells were kept under the same conditions, including transportation, but were not exposed to radiation or the aptamer.

### 2.7. Transcriptome Analysis

G01 glioblastoma cells were treated with the bi-(AID-1-T) aptamer alone, with irradiation alone, or with a combination of the two, as described above. For transcriptome analysis, the cells were treated with Trizol reagent (Thermo Fisher Scientific, Waltham, MA, USA) according to the protocol. The quality and quantity of the total RNA were assessed using a Bioanalyzer and an RNA 6000 Nano Kit (Agilent Technologies, Waldbronn, Germany). Then, the polyA fraction was isolated from the total RNA using Dynabeads^®^ mRNA Purification Kit oligoT magnetic beads (Ambion, Austin, TX, USA; Thermo Fisher Scientific, Waltham, MA, USA) according to the kit protocol. PolyA RNA libraries were generated using the NEBNext^®^ RNA Ultra II Kit (New England Biolabs, Hitchin, UK). The libraries were quantified using the Qubit dsDNA HS Assay Kit (Thermo Fisher Scientific, Waltham, MA, USA) on a Qubit 2.0 instrument and analyzed for fragment length distribution using the Agilent High Sensitivity DNA Kit (Agilent, Waldbronn, Germany). Sequencing was performed on a HiSeq 1500 instrument (Illumina, San Diego, CA, USA) with a minimum of 10 million 50-nucleotide short reads per sample. The following software was used to analyze differentially expressed genes:

- STAR (version 2.7.11) was used to index the GRCh38.p14 reference genome assembly (NCBI RefSeq GCF_000001405.40) and align the RNA-seq data.
- Gene expression levels were assessed using HTSeq (v. 2.0.5).
- Differential expression analysis was performed using DESeq2 (version 1.44.0).
- Over-representation analysis (ORA) for gene ontology terms was performed using the ClusterProfiler package (version 4.12.0) in R.

### 2.8. Statistics

A statistical analysis (ANOVA test) was performed using GraphPad Prism 9 software. The data are presented as means ± SD from a minimum of three repeats. For immunocytochemistry, data are presented as boxplots, with whiskers for min to max values and a bar as the mean. Statistical significance is indicated by the following: * *p* < 0.05, ** *p* < 0.01, *** *p* < 0.001, and **** *p* < 0.0001.

## 3. Results

### 3.1. The Effect of Combined Radiotherapy and bi-(AID-1-T) Aptamer Treatment on the Proliferative and Migration Potential of High-Grade Human Glioma Cell Cultures

We analyzed the effect of radiation with a dose of 20 Gy, aptamer bi-(AID-1-T), and their combination on 10 cell cultures of human high-grade glioma: five cultures of primary tumors and five cultures of recurrent gliomas (Table 1). All cell cultures were obtained from postoperative material of patients at the Federal State Autonomous Institution N. N. Burdenko National Medical Research Center of Neurosurgery of the Ministry of Health of the Russian Federation, and the cultures under study have already been used in previous studies [34,35].

The choice of a single radiation dose of 20 Gy in this study is based on modern approaches to modeling radiation exposure to glioma cells in vitro. In clinical practice, doses of 15–24 Gy are successfully used in stereotactic radiosurgery for recurrent glioblastoma [44,45]. In experimental in vitro studies, a dose of 20 Gy has proven effective in inducing a significant number of DNA double-strand breaks and subsequent activation of cellular damage response systems [46,47].

The choice of a 72 h time interval between irradiation and aptamer addition is necessary to ensure the full development of the cellular response to radiation damage. This approach allows for the evaluation of not only the immediate effects of irradiation but also the impact of combined exposure on key functional characteristics of cells, including proliferation and migration activity [47,48].

**Figure 1:**
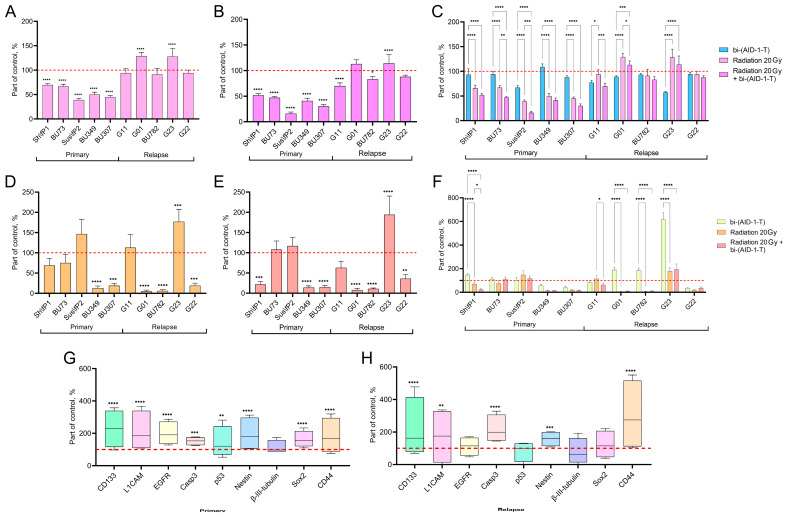
(**A**–**C**)—Effect on proliferative activity. Proliferation was assessed using the MTS assay 72 h after aptamer treatment. Data are presented as a percentage of the untreated control (100%, dotted line), as mean ± SD (*n* = 5). (**A**)—Cells were treated with a single dose of radiation (20 Gy). (**B**)—Cells were treated with radiation (20 Gy) followed by the addition of the bi-(AID-1-T) aptamer (10 μM). (**C**)—Comparison of three conditions: aptamer alone (10 μM), radiation alone (20 Gy), and the combination of radiation and aptamer. (**D**–**F**)—Effect on migration activity. Migration ability was assessed using a transwell assay. Data are presented as percentage of untreated control (100%, dotted line) migration, as mean ± SD (*n* = 3). (**D**)—Effect of a single dose of radiation (20 Gy). (**E**)—Effect of a combination of radiation (20 Gy) and the bi-(AID-1-T) aptamer (10 μM). (**F**)—Comparative analysis of the three conditions (aptamer, radiation, and combination). (**G**,**H**)—Combined effect of radiation and bi-(AID-1-T) aptamer on tumor marker expression. Staining was performed with antibodies against CD133, L1CAM, EGFR, Casp3, p53, Nestin, β-III-tubulin, Sox2, and CD44. The graphs show the average marker expression values for a sample of cell cultures (*n* = 4) as a percentage of the control, control is taken as 100 percent (red dotted line). Data are presented as boxplots, with whiskers for min to max values and a bar as the mean. Each marker on the graph has its own color for ease of understanding. The names of the markers are indicated on the lower axis. (**G**)—Primary high-grade glioma cell cultures. (**H**)—Recurrent high-grade glioma cell cultures. For all quantitative data, a two-way ANOVA test was used in GraphPad Prism 9. Levels of statistical significance: * *p* < 0.05, ** *p* < 0.01, *** *p* < 0.001, and **** *p* < 0.0001.

Our data revealed that radiation suppressed the proliferative activity of all studied cell cultures of primary tumors. However, no antiproliferative effect was observed in the cells of relapsed cultures; on the contrary, stimulation of proliferation was noted in the cultures of gliomas G01 and G23 (Figure 1A).

Remarkably, the addition of the bi-(AID-1-T) aptamer to the culture medium of human glioma cell cultures after radiation enhanced the antiproliferative effect both in the cell cultures of primary gliomas and in the cultures of recurrent gliomas that have not previously responded to radiation. In the case of G01 and G23 glioma cell cultures, the aptamer reduced the stimulating effect of radiation (Figure 1B).

We found that the combined approach, using exposure to radiation and the bi-(AID-1-T) aptamer, has the best effect on cell cultures of primary gliomas, while the best antiproliferative effect on cell cultures of relapsed gliomas is achieved with monotherapy, i.e., the bi-(AID-1-T) aptamer without radiation (Figure 1C).

Analysis of the radiation effect on the migration activity of cell cultures of human high-grade malignant gliomas showed differences in the response depending on the status of the glioma (primary or relapse). Thus, in most cell cultures of primary tumors, radiation had no effect on migration activity or led to its decrease. The exception was the cell culture Sus\fP2, where an increase in migration was noted. At the same time, in cell cultures of relapsed high-grade malignant gliomas, G01, BU782, and G22, a significant suppression of migration activity was observed. However, in cultures G11 and G23, radiation caused the stimulation of migration (Figure 1D)

We demonstrated that treatment with the bi-(AID-1-T) aptamer after radiation enhanced the anti-migration effect in most of the studied cell cultures of high-grade human gliomas. Statistically significant suppression of migration activity was observed in 6 out of 10 cultures. The exception was the G23 relapse cell culture, where migration stimulation was observed, like in the experiments when only radiation was used. It is worth noting that Sus\fP2 and G11 cell cultures, the ones that have previously demonstrated increased migration activity after radiation, showed no increase in migration activity when the combination of radiation and bi-(AID-1-T) aptamer treatments was used. (Figure 1E).

In summary, there was a clear correlation between proliferating activity of the tumor cells and the origin of the tumor. However, we found no link between the tumor cell origin and the migration capability of the tumor cells. The best anti-migration effect on all cultures was demonstrated by the use of combination of radiation with the bi-(AID-1-T) aptamer. Bi-(AID-1-T) monotherapy, on the contrary, might stimulate the migration activity of the cells (Figure 1F).

Interestingly, when radiation was combined with the bi-(AID-1-T) aptamer treatment, the cells characterized by increased expression of the stemness factors CD133, L1CAM, EGFR, Casp3, p53, Nestin, Sox2, and CD44 had increased survival rate in primary gliomas (Figure 1G). In relapse cell cultures, combination therapy also resulted in a significant increase in survival rate of cells with high expression of CD133, L1CAM, Casp3, Nestin, and CD44 markers (Figure 1H).

In summary, our data demonstrate that surviving cell populations in both primary and recurrent glioma cultures might represent a treatment-resistant cell sub-population.

### 3.2. Transcriptome Analysis of Human Glioblastoma G01 Cell Culture After Exposure to 20 Gy Radiation and bi-(AID-1-T) Aptamer

**Figure 2:**
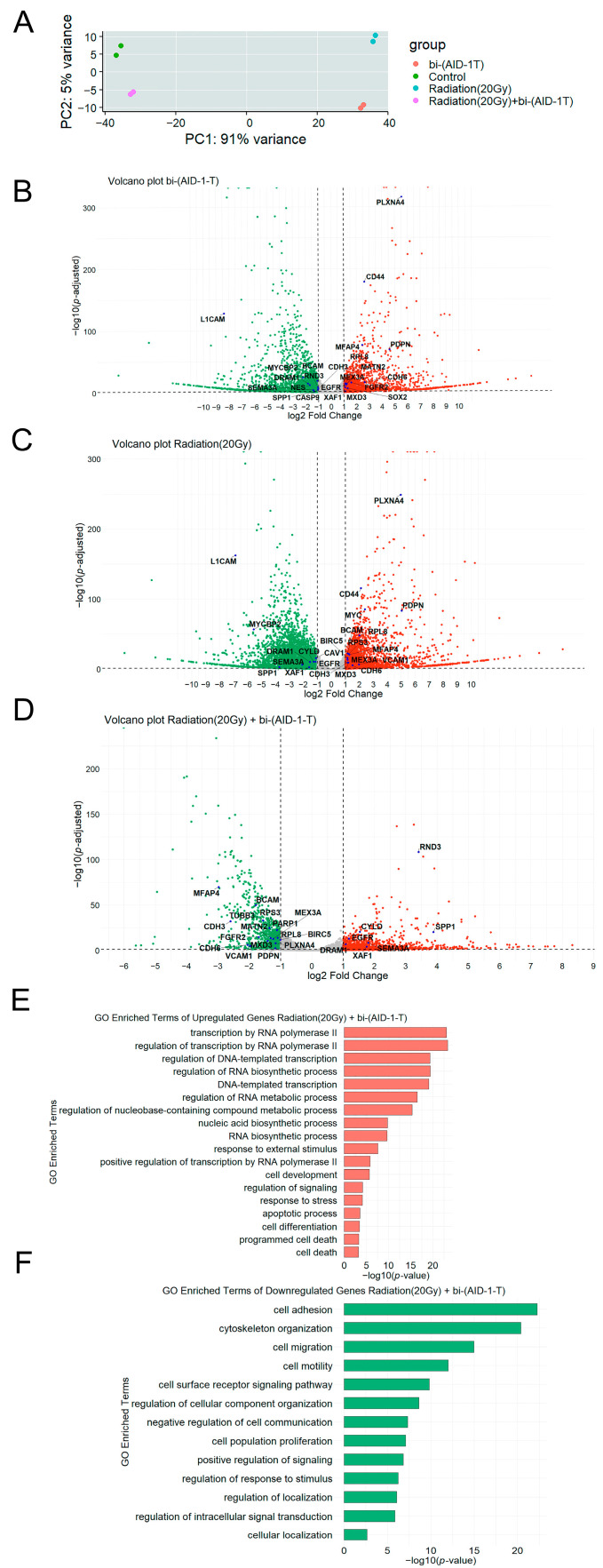
(**A**)—PCA plot displays general transcriptional profiles in control and experimental groups, consisting of four transcriptome analysis datasets of human glioblastoma G01 cells. Cells underwent either treatment with bi-(AID-1-T) aptamer (red), or exposure to radiation (blue), or combined exposure to radiation and bi-(AID-1-T) aptamer treatment (purple). Control cells are presented in green; (**B**)—volcano plot displaying differentially expressed genes (DEGs) after treatment of human glioblastoma G01 cells with bi-(AID-1-T) aptamer. DEGs (*p*-adjusted < 0.05) with log2 (fold change) greater than 1 are shown in red; DEGs with log2 (fold change) less than (−1) are shown in green. Insignificant genes are shown in gray. Horizontal line: −log10(0.05). Vertical lines: log2FoldChange value equal to −1 and 1; (**C**)—volcano plot displays differentially expressed genes (DEGs) after radiation of human glioblastoma G01 cells. DEGs (*p*-adjusted < 0.05) with log2 (fold change) greater than 1 are shown in red; DEGs with log2 (fold change) less than (−1) are shown in green. Irrelevant genes are shown in gray. Horizontal line: −log10(0.05). Vertical lines: log2FoldChange value equal to −1 and 1; (**D**)—volcano plot displaying differentially expressed genes (DEGs) after radiation of human glioblastoma G01 cells followed by bi-(AID-1-T) aptamer treatment. DEGs (*p*-adjusted < 0.05) with log2 (fold change) greater than 1 are shown in red; DEGs with log2 (fold change) less than (−1) are shown in green. Irrelevant genes are shown in gray. Horizontal line: −log10(0.05). Vertical lines: log2FoldChange value equal to −1 and 1; (**E**)—bar plot visualization of enriched GO terms for genes upregulated in human glioblastoma G01 cells after radiation followed by bi-(AID-1-T) aptamer exposure. The analysis was performed for comparison with the Biological Processes GO dataset; (**F**)—bar plot visualization of enriched GO terms of downregulated genes in human glioblastoma G01 cells after radiation followed by bi-(AID-1-T) aptamer exposure. The analysis was performed against the Biological Processes GO dataset.

Next, we investigated the effect of the bi-(AID-1-T) aptamer, 20 Gy irradiation, and combination of the radiation and aptamer on the transcriptome in human glioblastoma G01 cells. The results showed that all treatments caused significant changes in gene expression, which clearly separated the experimental and control groups in the principal component analysis (PCA) plot (Figure 2A).

After selecting genes with an average number of reads across samples greater than 10 and excluding long intergenic non-coding RNAs and designations for yet-uncharacterized genes from the list, 12,813 genes (26%) were selected from the total number of genes (50,037) for further analysis. After differentially expressed (DE) analysis, genes with an adjusted significance level *p* value < 0.05 and |log2FoldChange| value > 1 were selected.

In total, 1893 genes were upregulated, and 2241 genes were downregulated in the bi-(AID-1-T) treated sample, accounting for 45.7% and 54.3% of the total DE genes, respectively (Figure 2B).

We found that the expression of 2707 genes increased, while the expression of 3645 genes decreased in the samples that underwent radiation treatment, which accounted for 42.6% and 57.4% of the total number of differentially expressed genes (DEGs), respectively (Figure 2C).

Our data demonstrated that after the combined treatment (20 Gy radiation and bi-(AID-1-T) aptamer), the expression of 1164 genes increased, while the expression of 924 genes decreased, accounting for 55.8% and 44.2% of the total number of DEGs, respectively (Figure 2D).

To correlate the observed transcriptional changes in human glioblastoma G01 cells after radiation followed by the treatment with bi-(AID-1-T) with biological processes, we performed a Gene Ontology (GO) analysis. Since the most significant biological effect was observed after the combined treatment, i.e., radiation and aptamer treatment together, we focused mainly on the analysis of biological processes associated with gene expression after this treatment. Gene Ontology enrichment analysis of DE transcripts with increased expression shows an increase in the expression of genes involved in cellular biosynthesis processes, stress response, apoptosis, and programmed cell death (Figure 2E).

In turn, the enrichment analysis of Gene Ontology terms of DEGs transcripts whose expression is reduced shows a decrease in the expression of genes involved in the processes of cell migration, adhesion, proliferation and cell communication (Figure 2F).

We identified certain general patterns of proliferation and migration changes in G01 cell culture exposed to different treatment, i.e., aptamer, radiation, and combination of radiation and aptamer (Table 2). Thus, when exposed to both radiation and a combination of radiation with the aptamer, a decrease in the migration activity of the cell culture was observed. Proliferation was decreased when cells were exposed to the aptamer and a combination of the aptamer and radiation.

Based on our data, we suggest that by identifying common genes with decreased or increased expression in each group, we will be able to identify genes that might be involved in migration and proliferation processes, given that four groups of genes were identified for each process.

**Figure 3:**
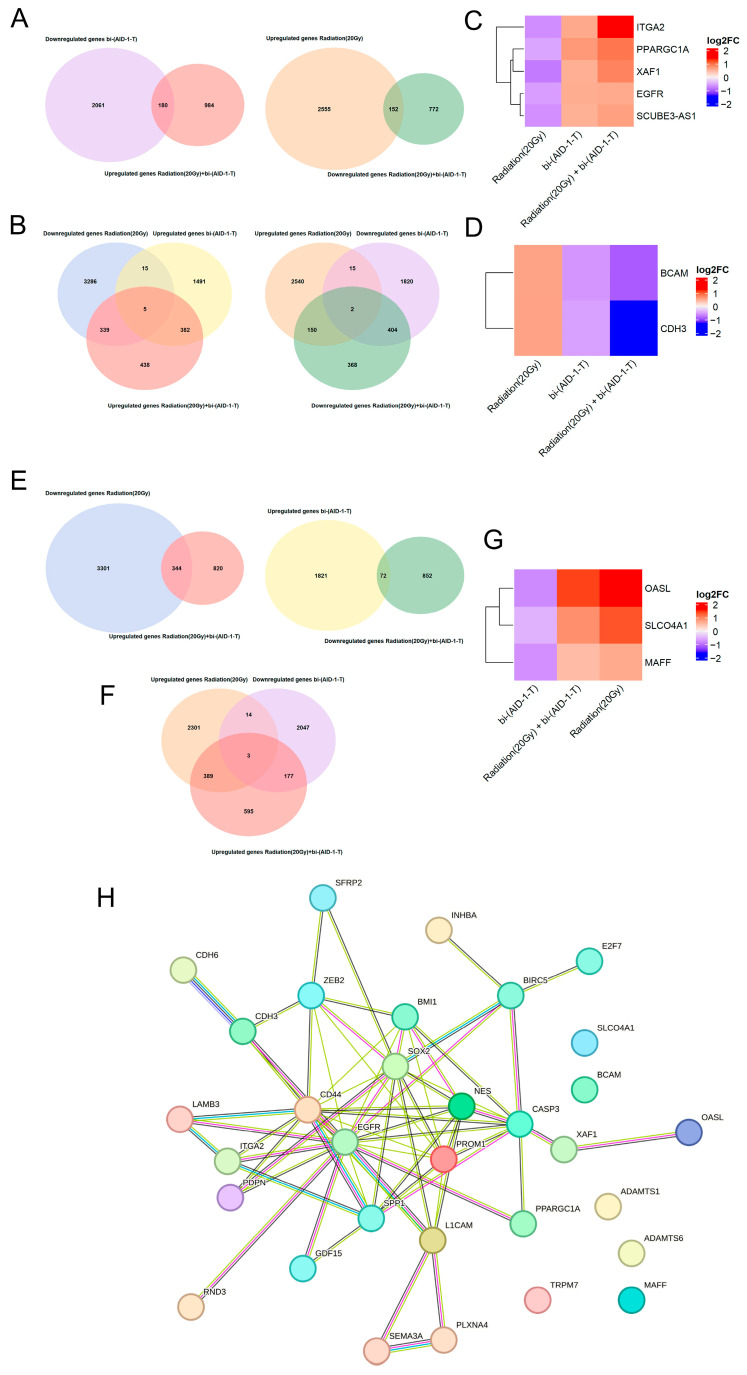
(**A**,**B**)—Venn diagrams showing the number of possible genes involved in the proliferation process. (**C**)—Heatmap analysis showing transcriptional changes in human glioblastoma G01 cells after treatment with bi-(AID-1-T) aptamer, 20 Gy radiation, and co-exposure to radiation and bi-(AID-1-T) aptamer and control (untreated cells). Overlap of gene groups downregulated by radiation with genes upregulated by aptamer and co-exposure to radiation and aptamer are shown. (**D**)—Heatmap analysis of transcriptional changes in human glioblastoma G01 cells after treatment with bi-(AID-1-T) aptamer, after 20 Gy irradiation, and after co-exposure to radiation and bi-(AID-1-T) aptamer. Control represents the cells without treatment. Overlap of the genes upregulated by radiation with genes downregulated by aptamer and co-exposure to irradiation and aptamer is shown. (**E**,**F**)—Venn diagrams showing the number of possible genes involved in migration processes. (**G**)—Heatmap analysis showing transcriptional changes in human glioblastoma G01 cells after treatment with bi-(AID-1-T) aptamer, radiation, and co-treatment of radiation with bi-(AID-1-T) aptamer. Control represents cells without treatment. Overlap of the genes upregulated by radiation and radiation combined with aptamer treatment and downregulated by aptamer alone is shown. (**H**)—Network of protein–protein interactions potentially involved in proliferation and migration processes. STRINGv12.0 was used to determine protein–protein interactions (PPIs) at the medium confidence level (CL = 0.400) (*p* < 1 × 10^−16^) (https://string-db.org).

To identify the genes that are potentially involved in proliferation process, we created an overlapping list of genes that decrease/increase upon radiation with genes that increase/decrease upon combined treatment. In addition, the overlapping analyses of genes that increase/decrease upon radiation with genes that decrease/increase upon exposure to the aptamer and the combined exposure to radiation and aptamer were generated (Figure 3A,B).

After we analyzed the overlap of the genes downregulated by radiation with genes upregulated by aptamer and combined radiation and aptamer, five genes were identified (Figure 3C).

One of the most interesting of these is the epidermal growth factor receptor (*EGFR*), a tyrosine kinase receptor that activates signaling pathways for cell proliferation and survival [49,50,51]. Its overexpression and mutations (including *EGFRvIII*) are associated with an unfavorable prognosis in glioblastoma [49,50,52]. The integrin *ITGA2*, which mediates adhesion and signaling, increases tumor aggressiveness when overexpressed, while its inhibition increases radiosensitivity [53,54,55,56]. *XAF1*, a regulator of apoptosis, promotes tumor cell survival when suppressed [57,58,59,60]. The coactivator *PPARGC1A* exhibits dualism. In hepatocellular carcinoma, it exhibits suppressive properties, while in other tumors, it can stimulate proliferation through metabolic reorganization [61,62].

Next, our heatmap analysis of the genes that were upregulated by 20 Gy radiation, downregulated by the bi-(AID-1-T) aptamer, and exposed to combined radiation and aptamer revealed two overlapping genes, namely *CDH3* and *BCAM* (Figure 3D).

*CDH3* (P-cadherin), a calcium-dependent adhesion molecule, in addition to maintaining tissue architecture, plays a role in regulating cell proliferation and differentiation. Aberrant *CDH3* expression is associated with an aggressive phenotype in various tumors, including gliomas, where its elevated levels correlate with a poor prognosis, likely due to its regulation of glial cell viability, invasion, migration, and stemness [63,64,65,66].

Another transmembrane receptor, *BCAM* (CD239), exhibits significantly increased expression in glioblastomas compared to normal brain tissue and low-grade gliomas, constituting an unfavorable prognostic factor. Its oncogenic activity is primarily mediated by binding to laminin-α5 in the extracellular matrix, leading to the activation of intracellular signaling pathways such as PI3K/AKT and MAPK/ERK, which directly stimulate the migration and invasion of tumor cells [67,68,69,70].

Based on the approach described above, we identified six genes that may be involved in the process of proliferation in glioblastoma cells. All of them, except *XAF1*, are constitutively overexpressed in tumors, including gliomas, and are known to be directly associated with the processes of proliferation and migration of tumor cells. Interestingly, when the cells were exposed only to the bi-(AID-1-T) aptamer or radiation together with the aptamer, a decrease in the expression level of *CDH3* and *BCAM* and an increase in the expression level of *XAF1* were observed. This may indicate a diminished aggressivity and migratory activity of the tumor cells after these treatments and possible initiation of apoptosis. However, at the same time, we also observed an increase in the expression level of *ITGA, EGFR,* and *PPARGC1A*, which may indicate the survival of an aggressive cell population.

A similar principle was used to identify genes potentially involved in the processes of tumor cell migration. Groups of genes that are increased/decreased by the aptamer and decreased/increased by the combined treatment of radiation and aptamer were analyzed. Also, groups of genes that are increased/decreased by the aptamer, decreased/increased by radiation, and the combined treatment of radiation and aptamer were analyzed (Figure 3E,F).

After analyzing the overlapping genes that are downregulated by the aptamer and upregulated by both radiation and the combination of radiation and aptamers, we found no common genes. However, after we analyzed the genes that are upregulated by radiation and the combination of radiation and aptamers but downregulated by the aptamer, three genes were found (Figure 3G).

The transcription factor *MAFF* is a member of the small Maf family of proteins, functioning primarily in heterodimers with CNC family factors (e.g., *NRF2*). It regulates gene expression by binding to antioxidant (ARE) or Maf-specific (MARE) promoter elements. *MAFF* plays a role in mediating the adaptive cellular response, maintaining redox homeostasis, and protecting against oxidative stress. However, despite its cytoprotective function, decreased *MAFF* expression is observed in various tumor types, and *MAFF* suppression has been shown to reduce metastatic potential, including in gliomas [71,72].

The OASL protein, known as a component of innate antiviral immunity, also plays a role in maintaining cellular homeostasis. Its interaction with the tumor suppressor *TP53* mediates the suppression of proliferation and the induction of apoptosis. Notably, *OASL* expression is often induced in response to DNA damaging agents [73,74,75,76]

In summary, we observed an increase in the expression level of *MAFF*, which may indicate the survival of more aggressive cells after radiation and the combined effect of radiation and the bi-(AID-1-T) aptamer. At the same time, an increase in *OASL* expression may indicate the activation of apoptotic processes.

Nevertheless, the obtained gene samples are too small to confidently state the effects of radiation with a dose of 20 Gy, the bi-(AID-1-T) aptamer, and their combination on any processes in cells, and a more in-depth analysis is needed on a wider sample of genes.

## 4. Discussion

Despite the extensive research, current standard treatment for patients diagnosed with glioblastoma and other high-grade gliomas involves a maximal surgical resection and postoperative concurrent radiotherapy in combination with chemotherapy. The protocol established by Stupp et al. demonstrated that the addition of temozolomide (TMZ) to fractionated radiotherapy (60 Gy, 2 Gy per fraction) statistically significantly increases median overall survival compared to radiotherapy treatment alone [77].

However, despite its effectiveness in improving survival rate, radiation therapy has significant drawbacks and is associated with serious side effects. The most serious unavoidable problem is the toxicity to the normal brain tissue, which leads to both acute effects and delayed health consequences for the patients [78,79].

Moreover, post-treatment radiation damage can be seen as a new focus of contrast enhancement on MRI, which may complicate clinical management of patients as it could be indistinguishable from true tumor progression (pseudoprogression) [80].

However, the most serious existing problem of the radiation treatment of gliomas is the development of therapy resistance and, as a result, inevitable relapse. These relapses are often highly aggressive and even more resistant to further treatments. It is well established that glioblastoma almost always recurs, usually within 2 cm of the edge of the primary tumor, falling into the zone of high-radiation doses [81,82,83].

These data strongly suggest that there is a subpopulation of tumor cells that survive the initial course of radiotherapy. The main subpopulation of cells that are considered to be the main source of these highly resistant cells is tumor stem cells (TSCs). TSCs have been shown to have significantly greater radio resistance in vitro and in vivo compared to their differentiated counterparts [17,84,85].

In addition, there is evidence that the therapy itself can selectively enrich the population of tumor-resistant cells and stimulate their aggressive behavior. Experimental studies have demonstrated that TMZ therapy increases the proportion of cells with stemness markers both in vitro and in xenografts [86].

Numerous studies have demonstrated that radiation therapy resulted in stimulation of tumor cell proliferation, migration, and invasion. It is plausible that radiation induces epithelial–mesenchymal (EMT) and EMT-like phenotypic changes in glioma cells associated with increased invasive potential [9,87,88,89,90].

It is possible that the relapses represent a more infiltrative and diffuse phenotype when compared to the primary tumor, which significantly complicates their treatment [89,90,91].

Antitumor therapy uses a combination of various methods to achieve the desired result. The widely used combination is the use of antitumor drugs with radiation therapy. As the most frequently used drug, TMZ, is known to increase tumor resistance to therapy, a new approach to the treatments becomes urgently needed. A promising alternative to the drugs may be aptamers—short oligonucleotides that can highly specifically bind to target molecules and are not toxic to healthy tissues.

Here, we investigated the use of G-quadruplex bi-(AID-1-T). This aptamer has previously shown promising results. demonstrating significant antiproliferative properties in primary gliomas. However, there was a negative effect on cultures of recurrent gliomas [35,41].

In this study, we aimed to understand whether radiotherapy, when used in combination with the G-quadruplex bi-(AID-1-T) aptamer, will improve the effectiveness of the aptamer treatment alone.

Our data revealed that radiation has a negative effect on the proliferation and migration of human glioma cell cultures. An increase in proliferative activity was observed after radiation with a single dose of 20 Gy in relapse cell cultures. At the same time, increased migration was noted in both relapse and primary glioma cultures.

Bi-(AID-1-T) aptamer treatment performed after 20 Gy radiation led to an increase in the antiproliferative effect on primary glioma cultures and reduced the negative effect of radiation in relapse cultures. Moreover, in primary gliomas, the antiproliferative effect was more pronounced when compared to the effect of aptamer-only treatment. Interestingly, in relapse cultures, however, the best antiproliferative effect was observed when the bi-(AID-1-T) aptamer was used alone, without radiation.

Having studied the migration effect, we found that in all cell cultures, regardless of the type of initial tumor, the best antimigration effect was observed when a combination of radiation and the bi-(AID-1-T) aptamer treatment was used.

However, we observed a significant increase in the expression of stemness and malignancy markers such as CD133, L1CAM, Casp3, Nestin, and CD44 in surviving cells of both primary glioma and relapse cultures.

CD133 is the main marker used to identify cancer stem cells (CSCs) in gliomas, particularly in the most aggressive form, glioblastoma. CD133-positive cells exhibit increased tumorigenic potential in experimental models and are capable of self-renewal and differentiation. They can reproduce the original tumor heterogeneity and are also associated with resistance to radiotherapy and chemotherapy [84,92,93].

The transmembrane glycoprotein L1CAM drives migration and invasion in gliomas. Interacting with cellular receptors activates signaling cascades involving SRC and FAK kinases, leading to cytoskeletal reorganization and increased tumor cell motility [94,95,96,97,98].

Caspase-3, a central effector of apoptosis, often functions differently in gliomas. Rather than leading to cell death, its sublethal activation can stimulate the proliferation of surviving cells, contributing to tumor resistance [99,100].

Expression of Nestin, a progenitor cell marker, correlates with the grade of glioma malignancy and is involved in the regulation of their growth, migration, and invasive potential [101,102,103].

The transmembrane glycoprotein CD44 plays a significant role in glioma pathogenesis, mediating both matrix adhesion and activation of signaling pathways that control invasion [104,105].

The fact that we observed the increased expression of these markers after combined radiation and bi-(AID-1-T) aptamer treatment may indicate the survival of the most aggressive and resistant cell population, which may lead to further relapses.

For a more in-depth understanding of the processes occurring after each treatment, transcriptome analysis of the G01 cell culture was performed after (a) treatment with bi-(AID-1-T) aptamer, (b) radiation, and (c) combined radiation and bi-(AID-1-T) treatment.

We identified DEGs after the combined treatment (radiation and the bi-(AID-1-T) aptamer) that are known to be involved in proliferation (*ITGA2*, *XAF1*, *EGFR*, *PPARGC1A*, *CDH3*, and *BCAM*) and migration (*MAFF*, *SLCO4A1*, and *OASL*). Most of the identified genes are constitutively overexpressed in tumors, including gliomas. After the combined treatment (radiation and bi-(AID-1-T) aptamer), a decrease in the expression of *CDH3* and *BCAM* genes and an increase in *XAF1* and *OASL* genes were observed, which may indicate a decrease in aggressiveness, migration, and initiation of apoptosis. However, a simultaneous increase in the expression of *ITGA*, *EGFR*, *PPARGC1A*, and *MAFF* and *SLCO4A4* genes indicates the possible survival of an aggressive subpopulation of cells after the combined treatment.

If we consider a wider range of DEGs after the combined treatment, additional genes such as *BMI1*, *BIRC5*, *E2F7*, *SPP1*, *GDF15*, *ZEB2*, and *SFRP2*, which are known to be involved in cellular proliferation processes, can be included in the analysis.

Analysis of the presented genes reveals several common molecular mechanisms underlying glioma pathogenesis. These genes are persistently overexpressed in tumor tissue compared to normal brain tissue, and the level of expression of each gene positively correlates with the grade of malignancy and predicts adverse clinical outcomes.

Factors such as *BMI1* and *BIRC5* directly counteract apoptosis and support cell survival. *BMI1*, a component of *PRC1*, additionally regulates cell self-renewal, while *BIRC5* inhibits effector caspases [106,107,108,109,110,111]. Oncogenic activity is enhanced by stimulation of proliferation and invasion mediated by *E2F7* and *SPP1*, where *SPP1*, by interacting with integrins and *CD44*, activates intracellular signaling pathways [112,113,114,115,116]. The invasive phenotype is further potentiated by the transcription factor *ZEB2*, which initiates epithelial–mesenchymal transition, and *GDF15*, which also promotes microenvironmental remodeling and metabolic reprogramming [117,118,119,120,121,122]. An important aspect is the modulation of signaling pathways, exemplified by *SFRP2*, which, despite inhibiting the canonical Wnt pathway, stimulates glial cell migration and invasion [123,124].

Thus, these genes collectively determine tumor resistance to therapy, suppression of programmed cell death, uncontrolled proliferation, and active invasion and, overall, contribute to the aggressive phenotype of gliomas.

To use a broader range of DEGs in the analysis, we added the genes from the inter-sections of groups shown in Figure 3A,E. As a result, a few additional genes known to affect migration were considered: *PDPN*, *TRPM7*, *LAMB3*, *SEMA3A*, *PLXNA4*, *RND3*, *INHBA*, *ADAMTS1*, *ADAMTS6*, and *CDH6*.

*PDPN* (Podoplanin) promotes an invasive phenotype through activation of the Wnt/β-catenin pathway [125,126]. *TRPM7*, essential for Mg^2+^/Ca^2+^ ion homeostasis, regulates cell adhesion and spreading [127,128,129]. *LAMB3* activates integrin-dependent FAK/Src pathways, enhancing migration and survival [130,131]. In contrast, *SEMA3A* and *RND3* act as invasion suppressors by inhibiting Rho/ROCK-mediated motility [132,133,134,135]. The *INHBA* subunit exhibits increased expression, which correlates with malignancy [136,137,138]. *ADAMTS1* functions dualistically: it can suppress tumor growth but also potentially support invasion via the Notch1-SOX2 pathway, whereas *ADAMTS6* has unambiguously pro-oncogenic properties [139,140,141,142]. *CDH6*, in turn, is essential for maintaining proliferation, invasion, and angiogenesis [64,143,144]. Thus, these genes form a complex network that coordinates the aggressive, invasive phenotype of gliomas.

It is noteworthy that the most frequently described genes are interconnected (Figure 3H) and can influence each other.

Our transcriptome analysis revealed that combination treatment with radiotherapy and the bi-(AID-1-T) aptamer induces a complex molecular response in high-grade glioma cells, characterized by the simultaneous activation of both anti-tumor and pro-survival pathways. Although we observed the expected therapeutic effects, including decreased expression of pro-proliferative genes (*PDPN*, *CDH6*, *CDH3*, *BCAM*, and *SFRP2*), increased expression of migration suppressors (*SEMA3A* and *RND3*), and induction of pro-apoptotic factors (*XAF1* and *OASL*), along with downregulation of the anti-apoptotic *BIRC5*, these beneficial changes were accompanied by increased expression of genes promoting migration (*ITGA2*, *MAFF*, *ZEB2*, and *TRPM7*) and proliferation (*EGFR*, *PPARGC1A*, *BMI1*, and *E2F7*). This apparent contradiction likely reflects the fundamental heterogeneity of high-grade gliomas and represents a common challenge in cancer therapy. We hypothesize that this dual response may be explained by several mechanisms. First, treatment may selectively eliminate therapy-sensitive populations while simultaneously enriching resistant clones that retain expression of aggressiveness markers. Second, surviving cells may activate compensatory pro-survival pathways as an adaptive response to therapeutic stress. Third, different tumor subpopulations may simultaneously undergo apoptosis, while others activate pro-survival pathways.

Our observed result, a significant reduction in both proliferation and migration, suggests that anti-tumor effects are dominant across the entire population. However, the persistent expression of pro-survival genes in a subset of cells may represent a potential mechanism for the development of therapy resistance and tumor relapse. This is consistent with clinical observations, where an initial therapeutic response is often followed by relapse caused by resistant subclones.

Further research should focus on single-cell resolution analysis to precisely identify cell subpopulations exhibiting these opposing molecular signatures, as well as on therapeutic strategies that specifically target resistant cells that retain expression of migration and proliferation promoters despite treatment. Further studies in more complex in vitro models are also needed to confirm these findings.

## Figures and Tables

**Table 1:** List of primary human glioma cell cultures obtained from patient postoperative material.

	Culture Name	Grade	Diagnosis
Primary tumor	Sh\fP1	4	Glioblastoma
	BU73	3	Anaplastic astrocytoma
	Sus\fP2	4	Glioblastoma
	BU349	4	Glioblastoma
	BU307	4	Glioblastoma
Relapse	G-11	3–4	Anaplastic astrocytoma
	G-01	4	Glioblastoma
	BU782	4	Glioblastoma
	G-23	4	Glioblastoma
	G22	4	Glioblastoma

**Table 2:** Changes in proliferation and migration of G01 cell culture under different treatment conditions.

	bi-(AID-1-T)	Radiation	Radiation + bi-(AID-1-T)
Migration	↑↑	↓	↓↓
Proliferation	↓↓	↑↑	↓

↓—a decrease in the parameter value; ↓↓—a strong decrease in the parameter value; ↑↑—a strong increase in the parameter value.

## Data Availability

The data presented in this study are available on request from the corresponding author. The data are not publicly available due to the reason that some of the generated data is not published.
